# Families with complex needs: an inside perspective from young people, their carers, and healthcare providers

**DOI:** 10.1007/s12687-022-00586-z

**Published:** 2022-03-18

**Authors:** Mădălina Radu, Ramona Moldovan, Adriana Băban

**Affiliations:** 1 Department of Psychology, Babeș-Bolyai University, Cluj-Napoca, Romania; 2 Division of Evolution and Genomic Sciences, School of Biological Science, University of Manchester, Manchester, UK; 3 Manchester Centre for Genomic Medicine, St Mary’s Hospital, Manchester University Hospitals NHS Foundation Trust, Manchester Academic Health Science Centre, Manchester, UK

**Keywords:** Complex needs, Young people, Carers, Professionals, Needs, Genetic counselling

## Abstract

Complex health needs are demanding and often require additional medical, psychological and social support. All those involved (e.g., patients, carers, professionals) face a unique set of challenges and needs, especially in families where the patient is a child or a young person with a lifelong condition. The aim of the study was to explore carers’ and young people’s needs when living with long term conditions, as well as the views of the healthcare professionals supporting them. Semi-structured interviews with 30 participants (11 professionals, 10 parents, and 9 young people) were conducted. Interviews focused on 3 main areas—(1) medical needs, (2) psychological needs, and (3) communication needs—and were analyzed using thematic analysis. Four main themes emerged: (1) *Acceptance takes time* refers to the often long and challenging process of adapting to the diagnosis and living with the condition; (2) *Close guidance* captures the importance of specialised and long term guidance in accessing and managing the complexities of the medical system; (3) *Open communication* shows the families’ need to be collaboratively connected to healthcare providers and other families facing similar difficulties; and (4) *Long-term support* underlines the importance of long term formal and informal support strategies. The participants’ experiences and views have provided us with insight and guidance for developing and implementing personalized and integrated services. This inside perspective is key for future research aimed at better understanding the impact of complex conditions and meaningful ways to support families throughout their journeys.

**Supplementary Information:**

The online version contains supplementary material available at 10.1007/s12687-022-00586-z.

## Introduction

The prevalence of complex health and psychosocial needs in families living with long term conditions is growing (McGregor et al. 2016). In healthcare settings, this is reflected in the multidimensional health and social care needs, both in the presence of a recognized medical condition or in the absence of a unifying diagnosis (Brenner et al. 2018). Complex needs most often involve additional medical, psychological, or social support; long-term personalized care; constant re-evaluation, adaptation, and management of care planning; involvement of several and often new stakeholders; accessing appropriate health, educational and community services; adapting family life according to each member’s needs (Brenner et al. 2018, 2021; McGregor et al. 2016). Recent research suggests that the impact of the unmet needs in this population may have been underestimated (McGregor et al. 2016; Smith et al. 2015). All stakeholders involved (e.g., patients, carers, healthcare professionals) face a unique set of challenges and needs. This is especially the case in families where the patient is a child or young person with lifelong conditions. Parents have to balance family dynamics, overcome financial difficulties, take care of their own needs and those of their children, advocate for adequate health care, often having to make decisions for and in the best interest of their children (Hill et al., 2018; Kasparian et al., 2018; McGrath et al. 2009; Patenaude and Schneider, 2017; Smith et al. 2015; Woodgate et al. 2015). Coordinating the healthcare framework with the family dynamic can be even more challenging when children and adolescents with complex needs transition into young adulthood (Bradford et al. 2018; Black et al. 2009; Slater et al. 2016; Woodgate et al. 2015). Young people’s needs and expectations vary significantly depending on their age and condition and require constant transitions, transformations and re-adaptations of care (Brenner et al. 2021; Kirk 2008). For example, young people have to adjust from co-care with parents to independence and autonomy in self-care, from pediatric to adult healthcare, from special education to special work conditions (Kirk 2008; Slater et al. 2016). Healthcare professionals are constantly searching for better ways to support families with complex needs (Bradford et al. 2018; Mattson and Kuo 2018) by developing appropriate communication strategies for both parents and young people (Bradford et al. 2018; Eisler et al. 2015; Hoang et al. 2018; Scollon et al. 2019) and developing or adapting services to appropriately meet families’ needs (Duncan and Young 2013; Gaff et al. 2006; Herman and Appelbaum 2010).

The needs of families living with complex conditions such as genetic disorders are not well documented in the context of emerging services, for instance multidisciplinary clinic genetic services and genetic counselling in countries like Romania where these pathways are currently being established (Abacan et al., 2019; Ciucă et al. 2021). In fact, very few studies have simultaneously addressed the perspective of all stakeholders involved in this process: parents, affected young people and healthcare professionals. The aim of the study was to explore carers and young people’s needs in the context of living with long term conditions as well as the views of the healthcare professionals supporting them.

## Method

### Participants

We conducted 30 semi-structured interviews using a data source triangulation method (Carter et al. 2014) to collect a comprehensive set of diverse experiences and to ensure data saturation. We interviewed 11 healthcare professionals, 10 parents, and 9 young people. Participants were recruited from clinical and special healthcare centers (e.g., Centre for Rare Diseases) in Cluj County, Romania. Purposive sampling was used to recruit the participants. The eligibility of the healthcare professionals was decided by the co-authors, based on their background and experience working with families with complex needs. The eligibility of the parents and young people was decided following conversations with various stakeholders involved in their care, based on their diagnoses or complex needs or their caregiver role. Participants were approached by the first author or a member of staff involved in their care, and were provided with details about the research project and the interviews. Potential participants were then contacted by the first author to discuss more details, sign the consent form and arrange the interview (e.g., face-to-face or telephone, date, time, and place). With the exception of 3 healthcare professionals (e.g., one general practitioner, two social workers) and 3 parents who were unable to take part in the research due to previous commitments, all those approached agreed to participate. Of note, two mothers suggested their daughters participate in the interview (e.g., parent 5 and young person 3; parent 7 and young person 7); the rest of people interviewed were not related. All carers were employed, with the exception of two mothers, one of which was on maternity leave. All young people interviewed were unemployed, but three of them (young persons 2, 8, and 9) were engaged in volunteer work with charities looking after individuals living with rare diseases. Participants’ characteristics are summarized in Table 1.

**Table 1:** Participants’ characteristics

Participant	Age	Gender	Young people’s diagnosis	Profession, years of experience (for professionals)
HP1 (health professional)	37	F	NA	Pediatric neurologist, 6 years
HP2	25	F	NA	Geneticist, 3 years
HP3	30	F	NA	Genetic counsellor, 8 years
HP4	45	F	NA	Child Psychologist, 5 years
HP5	29	F	NA	Child Psychologist, 5 years
HP6	28	F	NA	Genetic counsellor, 4 years
HP7	47	M	NA	Geneticist, 28 years
HP8	39	F	NA	Genetic counsellor, 8 years
HP9	32	F	NA	Child psychiatrist, 6 years
HP10	45	F	NA	Nurse, 22 years
HP11	26	F	NA	Psychologist from pediatric oncology, 4 years
P1 (parent)	38	F	Intellectual disability	Personal assistant
P2	46	F	Intellectual disability and epilepsy	Personal assistant
P3	38	F	Intellectual disability and ADHD	Administrative assistant
P4	38	F	Autism spectrum disorder	Unemployed
P5	43	F	Jacobsen syndrome	Personal assistant
P6	43	F	Down syndrome	Personal assistant
P7	55	F	Prader-Willi syndrome	NGO director
P8	51	M	Fragile X syndrome	Store manager
P9	41	F	Phenylketonuria	NGO manager
P10	28	F	Hydrocephaly, cerebral paralysis, and epilepsy	Maternity leave
YP1 (Young person)	35	F	Spinocerebellar ataxia	Unemployed
YP2	28	B	Hemophilia	Volunteer
YP3	19	F	Jacobsen syndrome	Unemployed
YP4	28	F	Intellectual disability	Unemployed
YP5	26	F	Intellectual disability and epilepsy	Unemployed
YP6	26	F	Down syndrome	Unemployed
YP7	33	F	Prader-Willi syndrome	Unemployed
YP8	34	F	Epilepsy and motor dysfunction	Volunteer
YP9	28	B	Achondroplasia	Volunteer

*NA* not applicable, *ADHD* attention deficit hyperactivity disorder

### Interviews

The interview guides were developed based on previous literature (Hines et al. 2009; Micheletto et al. 2013; Szybowska et al. 2007; von der Lippe et al. 2017) and were focused on 3 main areas: (1) medical needs (e.g., knowledge about diagnosis, previous experience with healthcare, type of services accessed); (2) psychological needs (e.g., coping strategies and social support); and (3) communication needs (e.g., communication with stakeholders; family dynamics and relationships) (see Appendix 1 for the interview guide). The interviews were conducted in Romanian by the first author (MR) between February and September 2018.

### Procedure

In-depth interviews were conducted individually, face-to-face (i.e., 11 healthcare professionals, 7 parents and 7 young people) or over the phone (i.e., parents 8, 9, and 10; young persons 10 and 11), in a private setting. No differences in terms of engagement or quality of the interaction were noticed between the two channels of communication. Before the interview, each participant had the opportunity to discuss the study at length and ask questions. All participants signed an informed consent form; where the participant had learning difficulties (i.e., young persons 3–7), the parent co-signed the consent form. Also, young people with learning difficulties and their parents were given the choice to attend the interview together, but all decided for the young person to attend alone. On average, the interviews had a duration of approximately 30 min. Interviews were audio-recorded and transcribed verbatim. The interviews had a similar structure, with questions adapted for each group. Ethical approval was obtained from the Ethics Committee at Babeş-Bolyai University.

### Data analysis

Thematic analysis was used to identify the main themes in the interviews (Braun and Clarke 2006; Carter et al. 2014). First, all interviews were transcribed and observations about the data were noted. Second, the interviews were coded in an inductive manner in order to extract relevant data. Third, the codes were color-coded and grouped based on color to facilitate theme searching. Fourth, when saturation was reached, interviews were repeatedly analyzed and recurring codes and themes were identified, named, discussed, and reviewed by all three researchers. Finally, quotes which eloquently reflected the main themes were selected and later were translated in English.

## Results

Following the thematic analysis, four main themes emerged: (1) *Acceptance takes time* refers to the often long and challenging process of adapting to the diagnosis and implications of a condition; (2) *Close guidance* captures the importance of specialized and long-term support in understanding and accessing the medical system and healthcare services available; (3) *Open communication* shows the families’ needs to be closely connected with healthcare providers and other families dealing with similar difficulties; and (4) *Long-term support* underlines the importance of long term formal and informal support strategies. Each theme is presented in detail below.

### Acceptance takes time

Healthcare professionals (HP) appeared to be well aware of parents’ difficulties to adapt to the diagnosis of their child. They believe the moment of diagnosis can be overwhelming; most often than not, it changes carers priorities and their day to day life. All professionals interviewed witnessed parents experiencing intense frustration, anger, guilt, shame, hopelessness, helplessness, and grief. Stigma was often mentioned as interfering with and prolonging the acceptance and adaptation process, for both parents and young people. Professionals often gave examples from parents about themselves or their children in various situations (e.g., playing in the park, interacting with other children in school, socializing with other parents, etc.) in which they felt rejected, judged, misunderstood or pitied. Healthcare providers pointed out that, in their experience, misconceptions about children and adolescents with complex needs often come from people who are not knowledgeable and do not fully appreciate the medical and psychological implications of various genetic conditions.

- HP10: “Many parents struggle with their children’s adaptation and integration in schools, for instance. They feel society is giving them a hard time.”

Many parents (P) said they needed time to come to terms with the condition. Most said that, at the moment of diagnosis, they felt frustration, anger, sadness, guilt, pain, uncertainty, and fear of the future. Some experienced shame and felt excluded from various social or educational activities because their child was “different.” Adaptation became easier after having contact with families in the same situation and after learning more about the condition, and how to manage it. If at the beginning carers were often overwhelmed, guilty or ashamed, with time they were able to overcome these feelings. Some parents mentioned their struggles helped them become closer and stronger as a family.

- P3: “I met many people along the way who were not at ease being around us, our child. There is this temptation to marginalize anyone who is different.”
- P7: “At the beginning I felt insecure. I was insecure because we did not know other people who were in the same situation as us. (…) But her diagnosis has had a great contribution to our human quality. She made us better (humans).”

Young people (YP) also mentioned needing time to accept their diagnosis and the implications that come with it. Almost all those interviewed said they experienced prejudice at some point in their life, especially during their teenage years, when they felt singled out or laughed at by peers for looking or behaving differently. Acceptance became easier after meeting other people with similar conditions or needs (people who have an “inside perspective”); it helped them manage depression, stigma and loneliness and made them feel connected and integrated.

- YP9: “I consider myself very much a normal person, but in school I was excluded, avoided, I was really discriminated against (…). The biggest influence was to meet people who have the same condition as me. In the city I live, I`m the only one and this made me feel depressed. But after I met them, it all became easier.”

### Close guidance

Professionals mentioned hearing parents say they could have used better guidance in understanding and adapting their expectations during the long and challenging process of finding the best care for their child. Accessing multiple medical sources (e.g., several doctors and hospitals) until the final diagnosis for their child and dealing with insufficient healthcare resources (e.g., medication, therapies, special schools, etc.) were some of the challenges mentioned. Professionals believed that the main reason carers do not always access the appropriate services is because they “get lost along the way.”

- HP1: “Parents have to fight for everything, from wheelchairs, to special health care, to special schools… everything.”
- HP9: “There is a great need for integrated services, a multidisciplinary clinic (…) I noticed that if a patient goes to one service, they no longer carry on the thread—they need 5 services but only access 2 services; because nobody guides them throughout the entire journey, many get lost.”

According to the professionals, one important source of guidance and support in this process is genetic counselling (GC). This service would facilitate a better understanding of and adaptation to the diagnosis and it would also help manage negative emotions (e.g., helplessness and guilt). Several professionals believed genetic counselling should be offered to all family members.

- HP3: “I think genetic counselling for these families might be a lifeboat. It would be that pillar, that source of support and information. Ideally before, but even right after a diagnosis, parents should access this service.”

Most parents had to wait a long time until a final diagnosis, sometimes one or several years. Carers mentioned having difficulties in understanding the process and navigating the health system, knowing where to look for information about the next steps or how to access the appropriate medical services. Parents mentioned that the ideal solution would be a one stop shop, a one stop service that could oversee their journey and guide them throughout.

- P3: “People are not informed in general… parents are not informed about all these things; there is no clear circuit for a child with needs that are different from the majority of children. There should be a counseling service… where you can go and ask questions, guidance.”

Parents also perceived genetic counselling as a service they could have benefited from. They believed it could have helped them understand the diagnosis, it could have facilitated decisions about family planning and informed healthcare strategies.

- P8: “I think we definitely needed genetic counselling right after we found out the diagnosis of our first boy. It would have been very useful from day one, because it would have helped us understand what we needed to do, to learn and to do differently.”

Young adults said they could have used genetic counselling when they were younger and started to have questions about their diagnosis. They believed it would have benefited both them and their parents in terms of information, guidance and long term support.

- YP3: “I already know all I need to know about my illness, but I think genetic counselling would have really helped my mum at that time (at diagnosis) or even before that, before having me or my brother.”
- YP1: “I talked once with a geneticist and she said something about recessive genes and my parents being carriers, but I don’t remember much now. I would be very interested in talking to someone about this again.”

## Open communication

Carers seemed to perceive professionals as the main gatekeepers of information about diagnosis, access to medical services and long-term care. All parents emphasized that a collaborative rapport and an open communication with professionals are key factors in their better understanding and addressing the needs of their child, as well as adapting their own expectations and efforts as caregivers.

- P10: “One of the therapists working with her always tells us exactly what she did and did not do, what she can and cannot do. This is very helpful and comforting to me because it helps me understand my daughter and her disease.”

When discussing communication as a family, parents see it very much as a long-term process, meaning that they tend to talk to their children about their condition gradually, in time.

- P7: “She knows she has Prader-Willi Syndrome. I tried to help her understand what she has, what she can and cannot do, what she should and should not do. I talk to her about the illness and I explain to her what that means. This is a continuous process.”

Several young people mentioned learning about their diagnosis when they were little, from their parents. Others said they did not talk to their parents about it, they rather learned about it in time, on their own, from books or from the Internet.

- YP1: “Yes, I talked with my parents… Not so much with my brothers, but with my parents I talk about my illness all the time.”
- YP3: “I did not necessarily have a conversation with them (parents). I started noticing things when I was 8 years old, when I was in the hospital and I met other children with hemophilia’… basically we didn’t have a conversation. I just got used to the illness.”

Professionals said they mostly discussed the condition with the parents. Most mentioned paying close attention to the parents’ needs and strongly believed an open communication is essential in establishing a good relationship with them. They believed not being open and transparent can negatively impact families and their trust in the medical system. Furthermore, professionals seemed to perceive carers as the main gatekeepers of information in the family, especially when the affected child is young. This is clearly a contrasting view to the one parents mentioned.

- HP11: “Doctors present information to parents as clearly as possible, information about diagnosis, treatment and management. It's important to get this information from the doctor.(…) After children become 18 years old they are treated in the adults section and things are different.”

## Long-term support

When asked about sources of resilience and support in their day to day life, almost all parents and affected youth said that the greatest support comes from other family members (e.g., partners, grandparents, aunts, uncles, siblings). They relied on the extended family for emotional, social, practical, and financial support.

- P5: “My sisters, my brothers, my mother, my parents, my relatives, my neighbors, they've always been there for me. My family has always been my support. They helped me morally and emotionally and I know I have them around me and I have whom to go to. And financially too, when I need it, they really do help me.”

Young people also mentioned that although they would like to meet new people, it is hard for them to do so. They also mentioned having childhood friends and developing close friendships with other young people who have the same diagnosis as them, as another meaningful source of support.

- YP1: “I do socialize, if I have who with. But I do not have so many people around me. My long-time friend comes and visits me often (…). You know, people often have this expectation: if I visit you, then you have to visit me too. And I can’t do that.”

Professionals believe families need constant and personalized support. They feel support groups, for instance, are a source of information on non-medical aspects, like education, career options, sexual education, romantic relationships etc.

- HP5: “They (parents) wondered about how friendships, romantic relationships and sex education are for people with Down syndrome. The problem was: Okay, how do we explain to our children who are adults, but they are adults with a certain IQ, what these basic needs mean, what sexuality means, how they can express it?”

## Discussion

Our study was aimed at exploring parents’ and affected young people’s needs in the context of living with long term conditions as well as the views of the healthcare professionals supporting them. We conducted 30 semi-structured interviews with 11 healthcare professionals, 10 parents, and 9 young people and explored in depth their medical, communicational, and psychological needs. Four main themes were identified: (1) acceptance takes time, (2) close guidance, (3) open communication, and (4) long-term support.

Professionals believe one of the first struggles families with complex healthcare needs are faced with is acceptance of the diagnosis. All professionals mentioned parents tend to experience intense emotions like anger, frustration, hopelessness, helplessness, grief, shame, and guilt, which were perceived as being part of the acceptance process. Stigmatization was seen as a major obstacle in this process, as it limits social interactions and impacts well-being, all of which increase parental stress and burden of care (Jenerette and Brewer 2010; Rani and Thomas 2019). Parents and young people also talked at length about acceptance as a long-term process. Receiving a diagnosis was only the first step in their long journey (Baumbusch et al. 2018); most parents mentioned struggling with intense emotions, and how they learned how to cope with them for many months and often years after the diagnosis. Some parents found it somewhat easier to come to terms with the diagnosis after accessing healthcare support, others after meeting families who were going through something similar; these families were seen as “having an inside perspective,” mainly because they understood first hand the complexity, the practical and psychological challenges their healthcare and familial needs involved. Similar to other studies (Baumbusch et al. 2018; Jamieson et al. 2014), having contact with other patients and families with complex needs was seen to facilitate acceptance, adaptation, exchange of information, and coping strategies as well as to promote social connections and inclusiveness.

After the diagnosis, the main responsibility for addressing all medical needs was often shared by both healthcare professionals and carers. Several examples were given during the interviews about each family’s journey to accessing and managing the complexities of the medical system. Parents had to balance several roles, such as “case manager, student, teacher, detective, guard and advocate” (Woodgate et al. 2015). This “intense parenting” (Woodgate et al. 2015) can have long-term consequences on carers physical, psychological, and emotional health (Caicedo 2014; Jackson et al. 2016; Martin and Nisa 1996; Woodgate et al. 2015). Parents and professionals believed specialized guidance would have helped them prepare for the responsibility of caregiving, by managing unrealistic expectations (e.g., fast diagnosis, straightforward access to medical resources) and reducing emotional distress (e.g., loss, helplessness, hopelessness) (Folkman and Greer 2000; Lipinski et al. 2006). Genetic counselling was perceived as a source of structured guidance. This process is aimed at helping families understand and adapt to the medical, psychological, familial, and reproductive implications of a specific health condition (Resta et al. 2006). It is a valuable source of knowledge about the medical condition, management, family planning, and resources available, as well as an important source of empowerment, which can guide parents in understanding and adapting to the idea of long term care (Athens et al. 2017; Caicedo 2014; Lipinski et al. 2006). Genetic counsellors can have a key role for these families before, during, and after the diagnosis (Bradford et al. 2018).

The theme “Open communication” captures the needs of parents and young people to be connected to a channel of information and communication about their health. Our results show, as did other studies (Dey et al. 2015), that the first and main source of information and guidance for parents are the professionals; they teach parents essential information about health literacy, management, long-term consequences, and how to communicate about it (Eisler et al. 2015; Maloney et al. 2012; Scollon et al. 2019; Tercyak et al. 2007). Parents sometimes saw the professionals as “gatekeepers,” and felt that the communication was not as open as they would have liked; interestingly, some professionals mentioned that sometimes parents take the role of “information gatekeepers,” probably due to the challenges in finding the appropriate language or timing at which they could explain the diagnosis to their children (Gallo et al. 2010; Metcalfe et al. 2011; Rivard and Mastel-Smith 2014). Most parents tried to tackle these challenges by talking about the diagnosis over a long period of time during childhood and adolescence, rather than addressing it all at once. Young people vividly remember having these conversations with their parents or reading information from books or the internet; few of them remember talking to healthcare professionals. Additional research exploring health care communication directly with young people is clearly required (Slater et al. 2016), as communication with parents alone has the potential of having a negative impact on young people with complex healthcare needs, by making them feel ignored, isolated or devalued (Jamieson et al. 2014).

The fourth core need, mentioned by all participants, was long-term support. Professionals talked about the importance of long-term professional guidance and specialized support groups to help families adapt to specific situations (e.g., labor integration, sexual education), (Housten et al. 2015; James et al. 2003). Young people focused less on specialized support, and mentioned their wish to have access to informal social groups and interactions. Health associated difficulties (e.g., walking outside, going to school or social events) often interfere with creating significant social bonds or maintaining friendships (Bethell et al. 2002; Jamieson et al. 2014; Sisk et al. 2018). However, both carers and young people mentioned the important help they have had from the extended family in their day to day life and in managing specific difficulties (e.g., financial problems, daily chores, etc.). One other aspect that some parents highlighted here was the role of siblings in sharing caregiving roles and supporting their families. Some parents also discussed the family dynamic they may or may not have always been aware of; some felt that for several years most of their attention was focused on the affected child and they may not have given the appropriate attention to the needs of their other children. The data available to us from the interviews was insufficient for a more in depth analysis of siblings’ perspectives, needs, and roles, but this is clearly a very important aspect to highlight, especially in the context of emerging research addressing this topic (Malcolm et al. 2013; Skotko et al. 2011).

Families living with complex conditions are in need of long-term support, both formal and informal, in all areas of their lives. As reflected in the interviews, the moment of the diagnosis was particularly challenging for most parents, as it became the beginning of a sinuous and unpredictable journey. While efforts to meet these needs are being made in all healthcare systems, services currently available clearly vary considerably from country to country. For example, genetic counselling has been consistently shown to improve knowledge, empowerment, emotional distress, risk perception, decision making, stigma, etc. (Athens et al. 2017; Madlensky et al. 2017), yet access and availability of this service is rather limited (McGrath et al. 2009; Shea et al. 2013; Smith et al. 2015; Wang and Watts 2007), with only 7000 genetic counsellors globally (Abacan et al. 2018), the majority of which working in North America and Western Europe. Given its multidisciplinary nature, genetic counselling can be key in supporting families impacted by genetic conditions throughout their journey, as it can help families with information and guidance which can improve their coping and acceptance process.

This study is not without its limitations. One important aspect that we need to acknowledge is that most parents interviewed were mothers. At the time of the interviews, four mothers were full-time caregivers of their children and two mothers worked in nongovernmental organizations looking after children with genetic or rare diseases. This may explain their interest to participate in this project and contribute with their personal experience. This is not dissimilar to other studies where mothers appear to be more involved in looking after children and adolescents with complex needs and also report on their experience (Adelman et al. 2014; Gérain and Zech 2019). Another aspect to be factored in when interpreting the results is the wide range of ages our young participants had. Clearly, the definitions of *young* vary considerably, and we opted for the most inclusive one. Results may however reveal a different view of their condition or needs if younger participants are interviewed.

By concatenating the views of affected young people, their parents and healthcare providers in the context of complex conditions, we have obtained an in-depth understanding of their experiences and needs. Following a diagnosis that is often unexpected, unpredictable or overwhelming, both families and professionals feel that close guidance, long-term support, and open communication contribute significantly to their adaptation and well-being. Genetic counselling, explicitly mentioned by some professionals or tentatively described by some parents or young adults, is an example of a service that can address these needs and contribute with the appropriate support for families living with complex conditions. These findings can provide guidance for developing and implementing more personalized and integrated services (genetic counselling being one example), as well as support future research aimed at better understanding the impact of complex conditions and meaningful ways to support families throughout their journeys.

## Supplementary Information

Below is the link to the electronic supplementary material.

Supplementary file1 (DOCX 20 KB)

## Declarations

All procedures followed were in accordance with the ethical standards of the Babeș-Bolyai University institutional research board, the responsible committee on human experimentation and with the Helsinki Declaration of 1975, as revised in 2000 (5).
