# Supplementary material for: Families with complex needs: an inside perspective from young people, their carers, and healthcare providers
Source: J Community Genet. 2022 Mar 18;13(3):293–302. doi: 10.1007/s12687-022-00586-z (PMC9270528; doi:10.1007/s12687-022-00586-z)
Supplement: Supplementary file 1 — Supplementary file1 (DOCX 20 KB) [file 12687_2022_586_MOESM1_ESM.docx]

**Interview guide**

**Healthcare providers**

(1) Medical needs

- Do you work with young people with complex needs? What are the main conditions you tend to work with?
- What can you tell me about what it’s like to work with families who have children with complex needs (e.g. prompt specific condition)?
- What are the most difficult aspects when working with families who have children with complex needs?
- What pathways or services do you generally recommend? What can you tell me about these services and how do you feel, in your experience, the families find them?

(2) Psychological needs

- What do you consider to be the main difficulties for these families (e.g. psychological, educational, practical etc.)?
- What do you consider to be their main needs (e.g. psychosocial, educational etc.)?
- What do you think are their main sources of support (e.g. medical, psychosocial etc.)?

(3) Communication needs

- How specific/detailed do you think professionals should be when discussing the diagnosis?
- Have you ever been asked by parents to talk about the diagnosis with their child? In your experience, is it something they prefer to discuss themselves, at their own pace? To what extent would you say parents prefer for you to bring it up/discuss it with their child?
- What do you consider to be the main communication/informational needs in these families (e.g. parents, young people)?
- What advice would you give to a young professional when working with families with young people with complex needs?

(4) Closing:

- Is there anything else you would like to add?

**Interview guide**

**Parents**

(1) Medical needs

- When was your son/daughter diagnosed? What was/is the diagnosis? How was he/she diagnosed (e.g. medical tests, genetic tests)? Can you tell me more about that time, that process?
- What were the pathways/services you had access to, at that time? Looking back, what services would have been useful?
- How would you describe your son’s/daughter’s condition, their day to day life.

(2) Psychological needs

- How would you say your son/daughter has adapted to his/her condition? How has it been for you personally, and your family?
- What would you say are, or have been, the most difficult issues you had to deal with?
- What are the main sources of support for you and your son/daughter (e.g. social, emotional, practical, medical, etc.)?

(3) Communication needs

- Who did you talk most with when trying to understand your child’s condition (e.g. doctor, friends, colleagues, teachers)? What was that like?
- Do you and your son/daughter discuss his/her condition? Have you discussed this in the past? Do you remember when the diagnosis was first mentioned and by whom? Were there follow-up conversations? Do you remember who prompted them?
- Where do you tend to look for information or someone to talk to (e.g. professional services, internet, doctors, social workers, other parents, etc.)?

(4) Closing

- Is there anything else you would like to add?

**Interview guide**

**Young people**

Note: with / without parents being present

(1) Medical needs

- Could you please tell me a bit about your condition? How would you describe it to a friend, for example? What is your life like, every day? What do you do every day, what would you like to do?
- Do you remember seeing a doctor when you were diagnosed, or about that time? What about a nurse? A psychologist or a counsellor? Do you remember what it was like?
- How are things now? Who is looking after you? Do you think you could use some extra help? What do you think would be good?

(2) Psychological needs

- What would you say tend to be the highs and the lows, on a day to day basis? What are the most difficult things you feel you have to deal with? What helps you the most?

(3) Communication needs

- Who did you talk to about your condition? Have you talked to your parents about it? What about other people? Can you give me an example of such a conversation?
- Could you use someone else to talk to about these things? Who would that person be, or what would the situation be like? Is there anything in particular you would like to know, or discuss?

(4) Closing:

- Is there anything else you would like to add?
